# GU81, a VEGFR2 antagonist peptoid, enhances the anti-tumor activity of doxorubicin in the murine MMTV-PyMT transgenic model of breast cancer

**DOI:** 10.1186/1471-2407-10-397

**Published:** 2010-07-30

**Authors:** Kristi D Lynn, D Gomika Udugamasooriya, Christina L Roland, Diego H Castrillon, Thomas J Kodadek, Rolf A Brekken

**Affiliations:** 1 Division of Surgical Oncology, Department of Surgery, UT Southwestern Medical Center, 5323 Harry Hines Blvd., Dallas, TX 76259, USA; 2 Hamon Center for Therapeutic Oncology Research, UT Southwestern Medical Center, 6000 Harry Hines Blvd., Dallas, TX 76259, USA; 3 Division of Translational Research, Department of Internal Medicine, UT Southwestern Medical Center, 6000 Harry Hines Blvd., Dallas, TX 76259, USA; 4 Advanced Imaging Research Center, UT Southwestern Medical Center, 6000 Harry Hines Blvd., Dallas, TX 76259, USA; 5 Department of Pathology, UT Southwestern Medical Center, 6000 Harry Hines Blvd., Dallas, TX 76259, USA; 6 Department of Pharmacology, UT Southwestern Medical Center, 6000 Harry Hines Blvd., Dallas, TX 75390, USA; 7 Department of Chemistry, The Scripps Research Institute, 130 Scripps Way, Jupiter, FL 33458, USA

## Abstract

**Background:**

Vascular endothelial growth factor (VEGF) is a primary stimulant of angiogenesis under physiological and pathological conditions. Anti-VEGF therapy is a clinically proven strategy for the treatment of a variety of cancers including colon, breast, lung, and renal cell carcinoma. Since VEGFR2 is the dominant angiogenic signaling receptor, it has become an important target in the development of novel anti-angiogenic therapies. We have reported previously the development of an antagonistic VEGFR2 peptoid (GU40C4) that has promising anti-angiogenic activity *in vitro *and *in vivo*.

**Methods:**

In the current study, we utilize a derivative of GU40C4, termed GU81 in therapy studies. GU81 was tested alone or in combination with doxorubicin for *in vivo *efficacy in the MMTV-PyMT transgenic model of breast cancer.

**Results:**

The derivative GU81 has increased *in vitro *efficacy compared to GU40C4. Single agent therapy (doxorubicin or GU81 alone) had no effect on tumor weight, histology, tumor fat content, or tumor growth index. However, GU81 is able to significantly to reduce total vascular area as a single agent. GU81 used in combination with doxorubicin significantly reduced tumor weight and growth index compared to all other treatment groups. Furthermore, treatment with combination therapy significantly arrested tumor progression at the premalignant stage, resulting in increased tumor fat content. Interestingly, treatment with GU81 alone increased tumor-VEGF levels and macrophage infiltration, an effect that was abrogated when used in combination with doxorubicin.

**Conclusion:**

This study demonstrates the VEGFR2 antagonist peptoid, GU81, enhances the anti-tumor activity of doxorubicin in spontaneous murine MMTV-PyMT breast tumors.

## Background

Breast cancer is the most frequently diagnosed malignancy in women in North America. Advancements in standard treatment regimens have improved the overall outlook for breast cancer patients in recent years, however, 40,000 women a year succumb to this disease, highlighting the need for better treatment modalities [1].

Angiogenesis, the development of new blood vessels from existing vessels, is required for tumor progression and metastasis [2]. For this reason, tumor angiogenesis has become an important target for cancer therapy [3,4]. Vascular endothelial growth factor (VEGF), a primary angiogenic growth factor in many tumor types, binds to and activates VEGFR1 and VEGFR2 [5]. VEGFR2 is the dominant angiogenic signaling receptor, while the function of VEGFR1 is less defined. As the angiogenic VEGF receptor, VEGFR2 has become a central target in developing anti-angiogenic therapies.

Bevacizumab (Avastin^®^, Genetech), which binds to VEGF and prevents VEGF from binding to both VEGFR1 and VEGFR2, was the first clinically approved anti-angiogenic therapy [6]. Bevacizumab was recently approved for the treatment of HER2/NEU-negative breast cancer in combination with chemotherapy, validating the use of anti-angiogenic therapy in this disease [7]. The clinical success of bevacizumab has amplified the number of anti-VEGF therapies being developed and tested. These therapies may specifically block VEGF, VEGFR1, or VEGFR2, or promiscuously block both VEGFRs as well as other receptor tyrosine kinases [8-11].

We have previously reported the development of a peptoid, GU40C4, that has promising anti-angiogenic activity both *in vitro *and *in vivo *[12]. GU40C4 significantly reduced VEGF-induced VEGFR2 phosphorylation in both PAE-KDR and HUVEC cells. Furthermore, GU40C4 significantly reduces VEGF-induced HUVEC proliferation [12]. GU81, a derivative of GU40C4, was developed to increase binding affinity and therefore *in vitro *and *in vivo *efficacy. Peptoids are closely related to peptides, however, peptoids (oligo-N-substituted glycines) are engineered for improved serum stability and cell permeability compared to peptides [13]. Peptoids differ from peptides by having the side chain ('R' group) placed on the amide nitrogen of the backbone.

In this study, we assess the *in vivo *efficacy of GU81, a derivative of GU40C4, in the MMTV-PyMT transgenic breast cancer model. The MMTV-PyMT model was chosen because tumor progression has been extensively analyzed in this model and closely mirrors the progression of human disease [14]. Based on our previous work with GU40C4 [12], we hypothesized that GU81 would control breast tumor growth both as a single agent and in combination with chemotherapy. However, our findings indicate that GU81 is not effective as a single agent in the MMTV-PyMT model of breast cancer, but combination with doxorubicin produces additive effects.

## Methods

### Production of GU81

GU81 was developed based on the identified 'minimum pharamacophore' [15] of GU40C4 and the complete development strategy will soon be published elsewhere.

### GU40C4, GU81 competition ELISA

96-well ELISA plates were coated with 1 ug/ml mouse VEGFR1/Fc and VEGFR2/Fc (R&D Systems, Minneapolis, MN) in sensitizing buffer (0.621 g NaHCO_3 _and 0.275 g Na_2_CO_3 _dissolved in 100 mL of ddH_2_O, pH 9.5) overnight at 4°C. Each well was washed with 3 × 200 μL phosphate-buffered saline (PBS) and blocked with 20% Aquablock (East Coast Biologics, North Berwick, ME). 50 μL of biotin-labeled GU40C4 (final concentration 75 nM) was added to each well in the presence or absence 50 μL unlabeled GU81 (final concentration 7.5 μM) for 2 hours at room temperature. Each well was washed with 3 × 200 μL in PBS. 50 μL HRP-conjugated strepavidin (Jackson Immunoresearch, West Grove, PA) was added at a 1:10,000 dilution in PBS for 1 hour at room temperature. The plate was then developed with HRP substrate and absorbance was measured at 450 nm.

### Fluorescence-based ELISA

White, clear-bottom 96-well plates (Corning Inc., Corning, NY) were coated with 1 μg/mL recombinant human VEGFR protein (R&D Systems) in sensitizing buffer (0.621 g NaHCO_3 _and 0.275 g Na_2_CO_3 _dissolved in 100 mL of ddH_2_O, pH 9.5) overnight at 4°C. Each well was washed with 3 × 200 μL of phosphate-buffered saline (PBS) and blocked with block buffer (Pierce, Rockford, IL). 50 μL GU81 (about 5 μM) was titrated in at a 4-fold dilution series. Wells were washed with 5 × 200 μL of PBS and fluorescence was measured at 520 nm using a plate reader (Fluostar Optima, BMG Laboratories, Durham, NC).

### VEGFR2 autophosphorylation assay

Experiments were conducted using PAE-KDR (Sibtech Inc., Brookfield, CT) and HUVEC (Sciencecell, Carlsbad, CA) cells. Cells were serum starved overnight and treated with increasing concentrations of GU81 for 15 min followed by 1.3 nM VEGF for 7 min (Invitrogen, Carlsbad, CA) or bevacizumab (Avastin™, Genentech, South San Francisco, CA) treated VEGF at 37°C. Cells were harvested with lysis buffer, and lysates were separated by SDS-PAGE and transferred to PVDF membranes. Membranes were probed with anti phospho-VEGF receptor 2 (Tyr1175, 19A10) or total anti-VEGFR2 primary antibodies (Cell Signaling Technology, Danvers, MA) and subsequently developed with appropriate HRP-conjugated secondary antibody (BioRad Laboratories, Hercules, CA) followed by enhanced chemiluminescent detection (Pierce).

### Tumor Model and Treatment

MMTV-PyMT/Fvb transgenic mice were obtained from the Jackson Laboratory (Bar Harbor, ME) and a colony was bred and maintained in a pathogen-free facility at UT Southwestern. All animal studies were performed on a protocol approved by IACUC at the University of Texas Southwestern Medical Center. Treatment with GU81 at 260 ug/day delivered via osmotic pump (total volume, 100 μl over 19 days, Alzet, Cupertino, CA) placed i.p. and doxorubicin (2 mg/kg once weekly i.v.) began when mice reached 6 weeks of age and continued for 19 days prior to animal sacrifice. Caliper measurements were made twice weekly and tumor volume was calculated using D × d^2 ^× 0.52, where D is the long diameter and d is the perpendicular short diameter.

### Histology and Immunohistochemistry

Control and treated MMTV-PyMT tumors were fixed in formalin, embedded in paraffin, sectioned, and stained with hematoxylin and eosin (H&E) by the Molecular Pathology Core Laboratory. Tissue was snap frozen in liquid nitrogen, embedded in OCT media, and sectioned. Sections were fixed in acetone, briefly air-dried and blocked with 20% Aquablock (East Coast Biologics) for 30-60 minutes. Primary antibodies were used at a final concentration of 5-10 μg/ml and include: rabbit anti-perilipin [16], rabbit anti-adiponectin [17], rabbit anti-phospho-histone-3 (Ser10) (Upstate, Lake Placid, NY), rabbit anti-cleaved caspase-3 (Asp 175) (Cell Signaling), rat anti-endomucin (Santa Cruz Biotechnology, Inc., Santa Cruz, CA), chicken anti-VEGF (Abcam, Cambridge, MA), and goat anti-F4/80 (Santa Cruz Biotechnology). Primary antibody was incubated on sections for one hour at room temperature or overnight at 4°C. Negative controls were performed by omitting the primary antibody. Following washes, the appropriate fluorophore conjugated secondary antibody was added (Jackson Immunoresearch). Fluorescent slides were cover-slipped using Prolong with DAPI (Invitrogen). Sections were examined on a Nikon E600 microscope and images captured with Photometrics coolsnap HQ camera using Elements Software (Nikon).

### Met-1 Cell Assays

Met-1 cells were plated in Dulbecco's Modified Eagle Media (DMEM) (Invitrogen) supplemented with 10% FBS and allowed to adhere O/N. Cells were then treated with serum-free DMEM in the presence or absence of 2.5 μM GU81. Tumor-conditioned media (TCM) was collected and RNA harvested using TRIzol (Invitrogen) according to the manufacturer's directions at 24, 48, and 72 hours post-treatment. The quality of RNA was evaluated using spectrophotometry. The cDNA used for subsequent PCR was made using iScript (Bio-Rad Laboratories). The expression of *VEGF *was analyzed by quantitative real-time PCR using assay on demand from Applied Biosystems (*Mm00437308_m1*). *GAPDH *(Applied Biosystems, 4352339E-0909032) assay-on-demand was used as an internal reference gene to normalize input cDNA. Quantitative real-time PCR was performed in a reaction volume of 20 μL of cDNA and each reaction was performed in triplicate. We used the comparative Ct method to compute relative expression values. TCM was diluted 1:5 prior to analysis for VEGF concentration using the Quantikine Mouse VEGF Immunoassay (R&D Systems).

### Statistics

Data were analyzed using GraphPad software (GraphPad Prism version 5.0 for windows; GraphPad Software, San Deigo, CA, http://www.graphpad.com). Results are expressed as mean ± SEM. Spearman rank correlations were used to assess the association between VEGF levels and macrophage infiltration. Data was analyzed by t-test or ANOVA and results are considered significant at p < 0.05.

## Results

### GU81 inhibits VEGF-induced activation of VEGFR2

**Figure 1:**
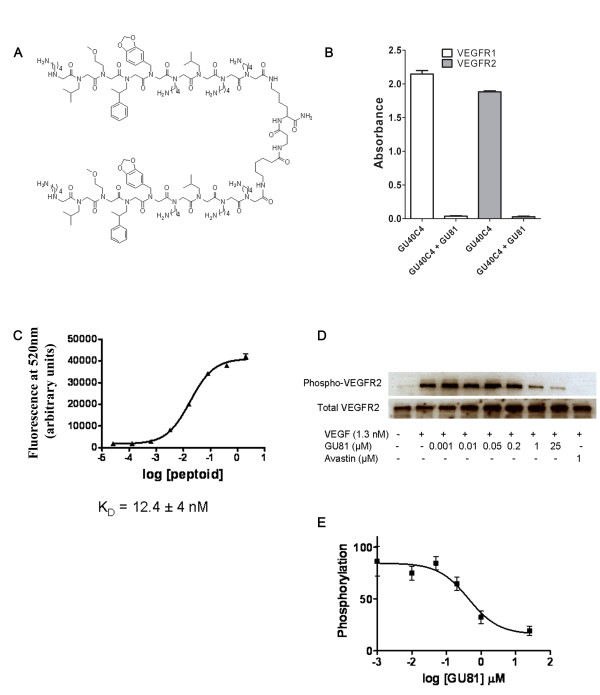
**GU81 inhibits VEGF-induced activation of VEGFR2**. A) Dimeric structure of the peptoid GU81 B) Biotin-conjugated GU40C4 binds to both VEGFR1 and VEGFR2 as measured by ELISA. GU81 competes with biotinylated GU40C4 for binding to both receptors. C) Fluorescein-conjugated GU81 was titrated and analyzed for binding to VEGFR2 as measured by ELISA. K_D_= 12 nM. D) Serum starved PAE/KDR cells were stimulated for 7 min with VEGF (1.3 nM) in the presence and absence of the indicated concentration of GU81. Avastin, a blocking anti-VEGF antibody was used as a positive control for inhibition. Cell lysates were made and separated by SDS-PAGE, transferred to PVDF membranes and probed for phosphorylated VEGFR2 and total VEGFR2 by Western blotting. E) Quantification and analysis of the results presented in (D). IC_50_= 430 nM.

GU81 (Figure 1A) is a derivative of the VEGFR2 antagonist GU40C4 [12]. We first characterized the ability of GU81 to compete with GU40C4 for binding to both VEGFR1 and VEGFR2 (Figure 1B). GU81 potently competes with GU40C4 for binding to both VEGFRs, indicating that these peptoids recognize the same epitope. We next analyzed the GU81 binding kinetics to VEGFR2 by fluorescence ELISA (Figure 1C). The measured binding constant for the GU81:VEGFR2 interaction (K_D_) was 12 nM, a 3-fold improvement over the original GU40C4 [12].

To validate the functional activity of the GU81, we conducted a VEGFR2 autophosphorylation assay [12]. In brief, PAE-KDR cells were grown, stimulated with VEGF and treated with increasing concentrations of GU81. VEGFR2 autophosphorylation levels were then detected by Western blotting (Figure 1D). We found that GU81 effectively blocks VEGF-induced VEGFR2 phosphorylation of PAE-KDR cells in a dose-dependent manner *in vitro*. Furthermore, through quantification of phospho-VEGFR2 and total VEGFR2 levels, we determined that the IC_50 _value for GU81 is ~430 nM. This is a 2-fold improvement over the parent compound GU40C4, which exhibits an IC_50 _value of approximately 1 μM [12]. For this reason, it is expected that GU81 is a more potent inhibitor of endothelial cell proliferation when compared to GU40C4, which inhibits VEGF-induced endothelial cell proliferation at a concentration of 1 μM [12].

### GU81 controls tumor growth in combination with doxorubicin

**Figure 2:**
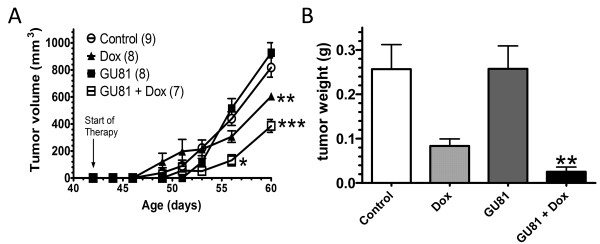
**GU81 improves that the anti-tumor efficacy of doxorubicin**. MMTV-PyMT mice (age 6 weeks) were treated 19 days with vehicle alone (control), doxorubicin (dox, 2 mg/kg 1×/week via ip injection in saline), GU81 (260 μg/day via osmotic pump), or GU81 + doxorubicin (GU81 + dox). A) Tumor volume is displayed as sum of total measurable tumor burden. *, p < 0.05; **, p < 0.01; ***, p < 0.005 vs control, 2-way ANOVA. B) Total tumor weight was calculated at time of sacrifice (age 60 days). Only GU81 + doxorubicin-treated tumors were significantly smaller than control treated tumors (p ≤0.01, 1-way ANOVA).

Following the *in vitro *validation of GU81 as an effective agent for blocking VEGFR2 autophosphorylation, we evaluated GU81 efficacy *in vivo*, as a single agent and in combination with doxorubicin in the MMTV-PyMT transgenic breast cancer model. Treatment began when mice reached 6 weeks of age and continued for 19 days prior to animal sacrifice. At the time of sacrifice, animals treated with either doxorubicin alone or GU81 + doxorubicin had significantly reduced tumor volume compared to control treated animals (Figure 2A). However, GU81 in combination with doxorubicin showed a striking additive effect. Tumor volume was significantly lower (1.6-fold reduction) in animals treated with GU81 + doxorubicin than those treated with doxorubicin alone (p ≤ 0.01, 2-way ANOVA) (Figure 2A). At time of necropsy all tumors were excised and weighed. Only animals treated with GU81 + doxorubicin had significantly less tumor burden (total tumor weight) compared to control treated animals (p ≤ 0.01, 1-way ANOVA) (Figure 2B). GU81 alone did not have a significant effect on either tumor volume or tumor weight (Figure 2A, B).

### GU81 alters tumor histology and the tumor microenvironment when used in combination with doxorubicin

**Figure 3:**
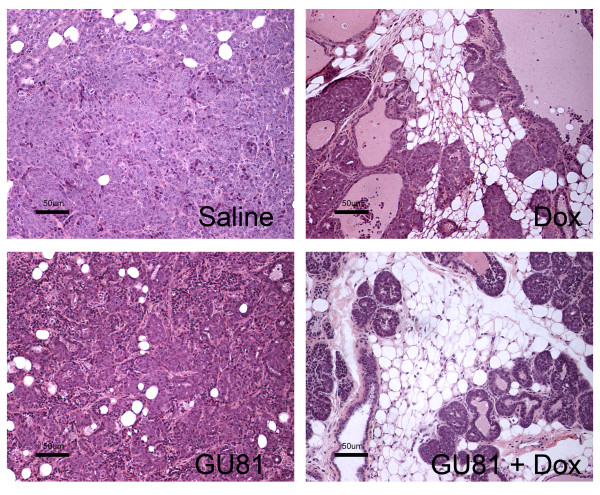
**Combination therapy of GU81 and doxorubicin prevents transition from a premalignant to an invasive phenotype**. Tumor tissue was harvested, fixed in formalin, sectioned, and stained with hematoxylin and eosin. Images representative of at least 3 tumors from each group are shown. Note the dramatic reduction in the number of invasive cells induced by combination therapy. Total magnification 200×; scale bar, 50 μm.

To evaluate the effects of therapy on the tumor microenvironment, hematoxylin and eosin staining was performed on formalin-fixed, paraffin embedded tumor sections. Tumors from saline-treated animals were classified as invasive carcinoma, and treatment with GU81 alone did not alter this histology (Figure 3). Tumors from doxorubicin treated animals also displayed areas of invasive carcinoma; although the invasive component of the tumor was reduced when compared to control and GU81-treated animals (Figure 3). In contrast, combination therapy significantly altered tumor/mammary tissue histology. In these animals, the mammary lobules were intact and there was no evidence of invasion. Consequently, more of the residual adipose tissue remained in the mammary gland (Figure 3). Thus, whereas control animals and animals treated with either GUI81 or doxorubicin alone harbored clearly invasive adenocarcinomas, the lesions in animals treated with combined GU81 and doxorubicin would be best described as hyperplastic or premalignant lesions, highlighting the marked additive effect of these agents (Figure 3).

**Figure 4:**
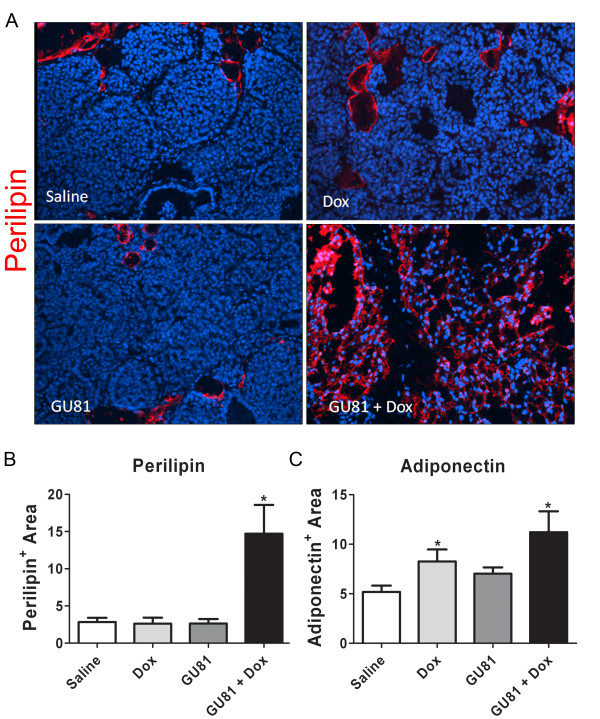
**Adipocyte structure is conserved in tumors from mice treated with GU81 and doxorubicin**. A) Sections of tumors from each treatment group were stained by immunofluorescence for perilipin (red), an adipocyte marker, and nuclei (blue). Images representative of each group are shown. A minimum of 3 tumors from each group were evaluated by immunofluorescence. Total magnification, 200×. B&C) Staining from a minimum of 5 images from each tumor per treatment group was quantified by evaluating the Cy3 positive area (perilipin(B) adiponectin (C)). There is significantly more perilipin (B) staining in tumors from mice treated with GU81 + doxorubicin than from tumors treated with either doxorubicin or GU81 alone (p≤0.05 by Mann-Whitney). Adiponectin (C) staining was significantly increased in tumors from mice treated with either doxorubicin alone or GU81 and doxorubicin compared to tumors from control-treated animals (p≤0.05, t-test).

To quantify the amount of normal breast tissue remaining in tumors from all treatment groups, immunohistochemistry was performed to evaluate the expression of the adipocyte markers perilipin and adiponectin (Figure 4). Perilipin coats lipid droplets in adipocytes and protects them against the body's natural lipases [18]. Adiponectin is secreted exclusively from adipocytes into the bloodstream where it regulates a number of metabolic processes including glucose regulation and fatty acid catabolism [19,20]. There were significantly higher levels of perilipin in tumors from GU81 + doxorubicin treated animals when compared to tumors from doxorubicin alone treated mice (p ≤ 0.01, 1-way ANOVA) (Figure 4B). Similar results were seen when immunohistochemistry was performed for adiponectin (Figure 4C) [19,21].

**Figure 5:**
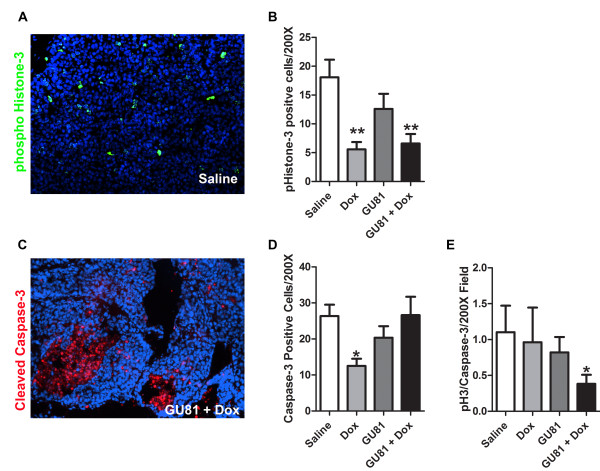
**Combination therapy of doxorubicin and GU81 reduces the growth index of MMTV-PyMT tumors**. Tumor sections from each treatment group were evaluated for phospho-histone-3 (A&B) and active-caspase-3 (C&D) by immunohistochemistry as described in the methods section. Signal intensity was quantified using Elements software and is displayed and mean +/- SEM. All quantification includes 3 animals/group and 5 sections/animal. D) A growth index was calculated whereby the number of phospho-histone-3 positive cells (actively proliferating) was divided by the number of active caspase-3 positive cells (undergoing apoptosis). *, p < 0.05; **, p < 0.01 vs control, 1-way ANOVA and Mann Whitney test.

Tumor growth dynamics were further evaluated using markers for phospho-histone-3, which stains actively proliferating cells, and cleaved caspase-3, which marks cells undergoing apoptosis. Tumors from animals treated with both doxorubicin alone and GU81 + doxorubicin had fewer phospho-histone-3 positive cells when compared to tumors from control treated animals, indicating that these tumors contained fewer actively proliferating cells (p ≤ 0.01, 1-way ANOVA) (Figure 5B). GU81 alone had no effect on tumor cell proliferation or apoptosis (Figure 5B, D). Interestingly, tumors from doxorubicin treated animals had significantly fewer active caspase-3 positive cells when compared to tumors from control-treated animals (p ≤ 0.05, 1-way ANOVA) (Figure 5D). The number of phospho-histone-3 positive cells/200× field was divided by the number of cleaved caspase-3 positive cells/200× field in order to obtain a growth index. A growth index of 1 indicates that the same number of cells are proliferating and undergoing apoptosis per field, whereas a growth index < 1 indicates that more cells are undergoing apoptosis than are proliferating. Only tumors from GU81 + doxorubicin treated animals had a significantly lower growth index compared to control treated tumors (p ≤ 0.05, 1-way ANOVA) (Figure 5E). These results show that whereas the single agent doxorubicin was cytostatic, combination therapy was cytocidal, consistent with the observed *in vivo *effects on tumor volume and weight.

### GU81 decreases total vascular area and vessel size but not vessel number

**Figure 6:**
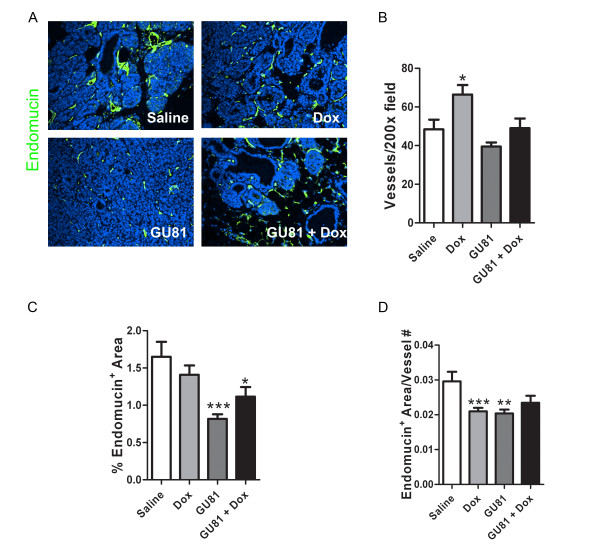
**Treatment with GU81 either alone or in combination with doxorubicin reduces total vascular area and vessel size in the MMTV-PyMT model**. Tumor sections from each group were evaluated for various vascular parameters following staining with the endothelial cell marker endomucin. Representative images from each treatment group are displayed in (A). (B) The number of vessels per 200× field was evaluated using Nikon Elements software and is displayed as the mean +/- SEM. All quantification includes 3 animals/group and 5 sections/animal. (C) The total vascular area was calculated using Nikon Elements software and is displayed as the percent fluorescent area per 200× field. (D) The percent fluorescent area was divided by the total vessel number for each section in order to obtain a relative vessel size. *, p < 0.05; **, p < 0.01 vs control; ***, p < 0.001, t-test.

To determine if GU81 had any effect on angiogenesis either alone or in combination with doxorubicin, microvessel density was assessed by immunohistochemical staining. Surprisingly, GU81 had no effect on total vessel number either alone or in combination with doxorubicin (Figure 6A, B). However, GU81 therapy decreased total vascular area, measured as the percent endomucin positive area per 200× field, alone (p < 0.001, t-test) and in combination with doxorubicin (p < .05, t-test) (Figure 6C). Furthermore, vessel size was decreased following all of the specified treatment regimens (Figure 6D).

### GU81 increases VEGF expression and macrophage infiltration, which is abrogated when used in combination with doxorubicin

**Figure 7:**
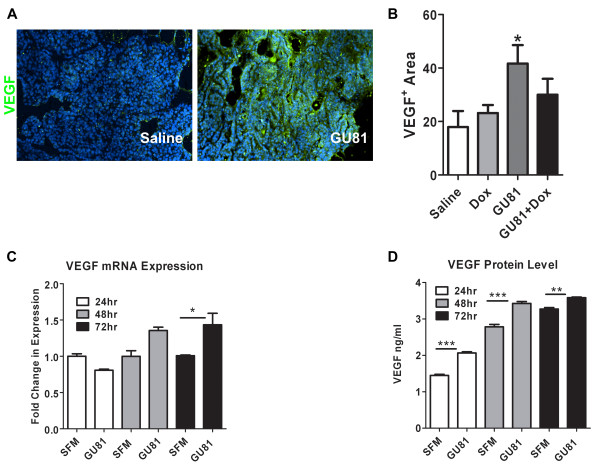
**GU81-alone but not in combination with doxorubicin induces VEGF expression *in vivo *and *in vitro***. Tumor sections from each group were evaluated for VEGF expression (A&B) using immunohisochemistry. The percent VEGF positive area was calculated using Elements software and is displayed as the mean +/- SEM. All quantification includes 3 animals/group and 5 sections/animal. * p < 0.05 vs control, t-test. C&D) Met-1 cells were treated with serum free DMEM +/- 2.5 uM GU81 for 24 hr, 48 hr, and 72 hr. mRNA and tumor conditioned media (TCM) was isolated and analyzed for VEGF levels at each time point. (C) Quantitative real-time PCR analysis reveals a significant increase in VEGF gene transcription following 72 hrs of GU81 treatment. * p < 0.05 vs SFM, 1-way ANOVA (D) VEGF protein levels were measured in Met-1 TCM using the Quantikine mouse VEGF ELISA kit (R&D Systems). VEGF secretion was significantly increased at all time points. ** p < 0.01, p < 0.001 vs SFM, 1-way ANOVA.

To further evaluate the tumor microenvironment following therapy, we assessed the levels of VEGF and F4/80^+ ^macrophage infiltration using immunohistochemistry. We found that tumors from GU81-treated animals had significantly increased levels of VEGF compared to tumors from control-treated mice (p ≤ 0.05, t-test) (Figure 7A, B). In contrast, neither treatment with doxorubicin alone or in combination with GU81 had significant effects on VEGF expression (Figure 6B). To further investigate the increase in VEGF expression following treatment with GU81, an *in vitro *system was utilized. Met-1 cells, which are a highly metastatic cell line derived from a MMTV-PyMT primary tumor [22], were treated in the presence or absence of 2.5 μM GU81 for 24, 48, and 72 hours. Following treatment, mRNA and tumor-conditioned media was collected for analysis of VEGF expression. *VEGF *mRNA levels are significantly increased after 72 hours of GU81 treatment (p < 0.05, 1-way ANOVA) (Figure 7C). Furthermore, VEGF protein levels were significantly increased at all time points analyzed following treatment with GU81 (p < 0.01, 1-way ANOVA) (Figure 7D).

**Figure 8:**
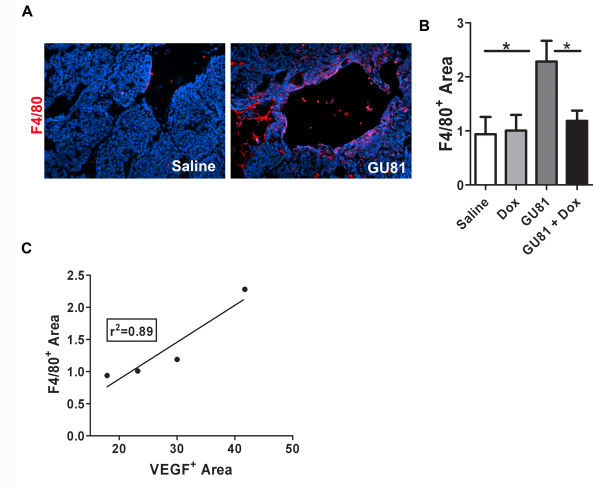
**GU81-alone but not in combination with doxorubicin induces macrophage infiltration *in vivo***. Tumor sections from each group were evaluated for macrophage infiltration by assessment of F4/80^+ ^cells (A&B) using immunohisochemistry. Representative images from saline and GU81 treated animals are displayed in (A). (B) The percent F4/80 positive area was calculated using Elements software and is displayed as the mean +/- SEM. All quantification includes 3 animals/group and 5 sections/animal. * p < 0.05 vs control, t-test. (C) Linear regression analysis reveals a positive correlation between VEGF levels and macrophage infiltration following therapy. r^2 ^= 0.89.

Macrophage infiltration into tumors is known to be influenced by VEGF [9,10,23]; therefore, we examined macrophage infiltration following therapy using the general macrophage marker F4/80. We found that macrophage infiltration was significantly increased following treatment with GU81 (p ≤ 0.05 vs. control-treatment, t-test) (Figure 8A, B). Furthermore, this effect was abrogated when GU81 was combined with doxorubicin (p ≤ 0.05 vs. GU81 alone, t-test) (Figure 6B). Linear regression analysis reveals a positive correlation between VEGF levels and macrophage infiltration following the described treatment regimens (r^2 ^= 0.89) (Figure 8C).

## Discussion

In this study, we describe the novel VEGFR2 peptoid antagonist, GU81. GU81 binds to VEGFR2 with a binding affinity of 12 nM and displays potent *in vitro *biological efficacy (Figure 1B&1C). We tested the therapeutic efficacy of GU81, both alone and in combination with doxorubicin in the extensively characterized spontaneous and aggressive MMTV-PyMT model of breast cancer [14]. While GU81 had little therapeutic efficacy when used alone, animals treated with the combination of GU81 and doxorubicin had decreased tumor burden (Figure 2A), significantly reduced tumor invasion (Figure 3), increased tumor fat content (Figure 4B), and a lower tumor growth index (Figure 5E) compared to animals from all other treatment groups.

We began therapy when mice reached 6 weeks of age, and at this time most of the mice harbored primary neoplasia that developed to the hyperplastic stage [14,24]. In this case, the combination of GU81 + doxorubicin was able to delay tumor progression and prevent transition from a premalignant to an invasive phenotype. This striking delay in tumor progression can be attributed to our use of a "double-edged sword" to target tumor cells (doxorubicin) and endothelial cells (GU81). In using this approach we were able to achieve significantly better results compared to either agent alone. Although we do not see a decrease in vessel number following treatment with GU81 either alone or in combination with doxorubicin, GU81 did induce a decrease in total vascular area and vessel size (Figure 6). Tumor blood vessels are dilated, leaky, and inefficient at delivering both oxygen and chemotherapeutic agents to the tumor [reviewed in [25]]. Vessel dilation is decreased following treatment with GU81, which leads us to the hypothesis that GU81 may be effectively normalizing the vasculature, decreasing hypoxia, and increasing doxorubicin delivery into the tumor.

Interestingly, GU81 does not demonstrate *in vivo *efficacy as a single agent in the MMTV-PyMT model of breast cancer [10]. This is consistent with our previous studies in the MMTV-PyMT model [10] and human data, which has shown that anti-VEGF therapy provides little clinical benefit as a single agent in breast cancer [26]. In contrast, GU40C4, the parent compound, demonstrated single agent efficacy in the A673 Ewings' sarcoma xenograft model [12]. GU81, however, does demonstrate *in vivo *efficacy as a single agent in the 4T1 syngenic breast cancer model, where it effectively reduces tumor size and MVD [10]. There are several possible explanations for the variation in response to GU40C4 and GU81. Most notable is the difference in tumor model systems. The MMTV-PyMT model is a spontaneous model that develops in the mammary fat pad, which is highly vascularized. While the A673 model is a subcutaneous xenograft model that is highly dependent upon VEGF activity [27], such that even low doses of anti-VEGF agents have a striking effect on tumor growth [28]. The 4T1 model is a syngeneic, highly metastatic inflammatory breast cancer model. Interestingly, recent work has identified VEGF as an autocrine survival factor for these cells under hypoxic conditions, which may explain the efficacy of GU81 as a single agent in this model [29]. It is possible that a higher dose given for a longer period of time would be more effective at reducing microvessel density in MMTV-PyMT tumors. Furthermore, it is important to highlight that treatment with GU81 alone increased tumor VEGF expression in this model, which may explain its inability to control tumor burden as a single agent (Figure 6B). We decided to further investigate this increase in tumor VEGF expression and confirmed our *in vivo *results in an *in vitro *system (Figure 7C, D). Tumor cells in primary MMTV-PyMT tumors express low levels of VEGFR2 (data not shown), however it is difficult to argue that either VEGF or GU81 may be having a direct effect on tumor cell proliferation or survival as we see no change in either proliferation or apoptosis markers following treatment with GU81 as a single agent. Met-1 cells also express VEGFR2 and increased VEGF expression following treatment with GU81 suggests that there may be an intact negative VEGF:VEGFR2 feedback loop in these cells. Treatment with GU81 could inhibit such a negative feedback loop, resulting in increased VEGF expression.

Macrophage infiltration is associated with poor prognosis in a number of different tumor types, including breast cancer [30,31]. After establishing that VEGF levels were increased following treatment with GU81 alone (Figure 6B), we decided to investigate what effect this increase has on macrophage infiltration, given that VEGF stimulates macrophage chemotaxis into the tumor microenvironment. GU81 increases macrophage infiltration as a single-agent [10], however, this effect is abrogated when GU81 combined with doxorubicin (Figure 6D). This increase in macrophage infiltration is puzzling as we and others have shown that anti-VEGF therapy can reduce macrophage infiltration in a number of pre-clinical models [9,10,23,32,33]. Additionally, we show that macrophages harvested from a tumor-bearing animal express both VEGFR1 and VEGFR2, whereas those harvested from non-tumor bearing mice are only VEGFR1^+ ^[9,23]. When VEGFR2 is expressed, it becomes the dominant receptor driving VEGF-induced chemotaxis and specific blockade of the VEGF:VEGFR2 interaction is sufficient to inhibit chemotaxis [9,23]. The most plausible explanation for the observed increase in macrophage infiltration may be attributed to the detected increase in VEGF expression and we have not ruled out the possibility that GU81 may reduce macrophage infiltration, if given at a dose that could compensate for the increased VEGF expression. We also cannot rule out the idea that another unidentified cytokine may be contributing to this increased macrophage infiltration following GU81 therapy in this model.

Future studies to determine the maximum tolerated dose (MTD) of GU81 in the MMTV-PyMT transgenic breast cancer model are currently underway. Once this has been established, mice will be treated at the MTD for longer periods of time to optimize the efficacy of GU81 both as a single agent and a combination therapy. Furthermore, we are interested in the mechanism by which GU81 increases VEGF expression and macrophage infiltration *in vivo *and are currently investigating this phenomenon.

## Conclusions

In conclusion, this study describes the use of a novel VEGFR2 antagonist peptoid, GU81, to effectively control tumor growth in combination with doxorubicin in a transgenic model of breast cancer that closely mimics human disease progression. This study further highlights the use of peptoids as new and exciting biological tools and potential therapeutics. Peptoids offer many desirable biological characteristics. They are easy to handle and inexpensive to synthesize and optimize. Furthermore, peptoids have high serum-stability [13], are non-immunogenic [34], and are cell permeable [35], thus making them good candidates for use in drug discovery.

## Competing interests

DGU, TJK, and RAB are listed on patents that describe GU81 and peptoids like it. These patents are owned by the University of Texas and licensed to Opko Health Inc for potential development of the technology.

## Authors' contributions

KL carried out the therapy study, performed and analyzed immunohistochemistry, and drafted the manuscript. DU designed and produced GU81, performed ELISA assay and *in vitro *autophosphorylation assay. CR participated in therapy study and tissue analysis. DC participated in analysis of H&E staining. TK participated in the design and coordination of the study. RB conceived the study, participated in its design and coordination and helped to draft the manuscript. All authors have read and approved the final manuscript.

## Pre-publication history

The pre-publication history for this paper can be accessed here:

http://www.biomedcentral.com/1471-2407/10/397/prepub
